# Use and Awareness of Heated Tobacco Products in Europe

**DOI:** 10.2188/jea.JE20200248

**Published:** 2022-03-05

**Authors:** Silvano Gallus, Alessandra Lugo, Xiaoqiu Liu, Elisa Borroni, Luke Clancy, Giuseppe Gorini, Maria José Lopez, Anna Odone, Krzysztof Przewozniak, Olena Tigova, Piet A. van den Brandt, Constantine Vardavas, Esteve Fernandez, the TackSHS Project Investigators

**Affiliations:** 1 Department of Environmental Health Sciences, Istituto di Ricerche Farmacologiche Mario Negri IRCCS, Milan, Italy; 2 The George Institute for Global Health, Sydney, Australia; 3 TobaccoFree Research Institute Ireland, TU Dublin, Ireland; 4 Istituto per lo Studio, la Prevenzione e la Rete Oncologica, Florence, Italy; 5 Agència de Salut Pública de Barcelona, Barcelona, Spain; 6 CIBER en Epidemiología y Salud Pública (CIBERESP) (Biomedical Research Centre Network for Epidemiology and Public Health), Spain; 7 Institut d’investigació Biomèdica Sant Pau (IIB St. Pau), Barcelona, Spain; 8 Department of Public Health, Experimental and Forensic Medicine, University of Pavia, Pavia, Italy; 9 IRCCS San Raffaele Scientific Institute, Milan, Italy; 10 The Maria Sklodowska-Curie National Research Institute of Oncology, Warsaw, Poland; 11 Collegium Civitas, Warsaw, Poland; 12 Foundation “Smart Health - Health in 3D”, Warsaw, Poland; 13 Tobacco Control Unit, Institut Català d’Oncologia, L’Hospitalet de Llobregat, Spain; 14 Tobacco Control Research Group, Institut d’Investigació Biomèdica de Bellvitge, L’Hospitalet de Llobregat, Spain; 15 Biomedical Research Centre Network for Respiratory Diseases for Respiratory Diseases (Centro de Investigación Biomédica en Red en Enfermedades respiratorias, CIBERES), Madrid, Spain; 16 School of Medicine and Health Sciences, Universitat de Barcelona, L’Hospitalet de Llobregat, Spain; 17 Department of Epidemiology, CAPHRI-School for Public Health and Primary Care, Maastricht University Medical Centre, Maastricht, The Netherlands; 18 Department of Epidemiology, GROW-School for Oncology and Developmental Biology, Maastricht University Medical Centre, Maastricht, The Netherlands; 19 Hellenic Cancer Society, Athens, Greece

**Keywords:** heated tobacco products, heat-not-burn tobacco products, IQOS, survey, Europe

## Abstract

**Background:**

Heated tobacco products (HTP) are new forms of tobacco consumption with limited information available on their use among the general population. Our objective was to analyze the prevalence and associations of use of HTP across 11 countries in Europe.

**Methods:**

Within the TackSHS Project, in 2017–2018 we conducted a cross-sectional study with information on HTP use in the following countries: Bulgaria, England, France, Germany, Greece, Italy, Latvia, Poland, Portugal, Romania and Spain. In each country, face-to-face interviews were performed on a representative sample of around 1,000 subjects aged ≥15 years, for a total of 10,839 subjects.

**Results:**

Overall, 27.8% of study participants were aware of HTPs, 1.8% were ever HTP users (ranging from 0.6% in Spain to 8.3% in Greece), and 0.1% were current users. Men were more frequently HTP ever users than women (adjusted odds ratio [aOR] 1.47; 95% confidence interval [CI], 1.11–1.95). Ever HTP use was inversely related to age (*P* for trend <0.001) and more frequent in ex-smokers (compared with never smokers, aOR 4.32; 95% CI, 2.69–6.95) and current smokers (aOR 8.35; 95% CI, 5.67–12.28), and in electronic cigarette past users (compared with never users, aOR 5.48; 95% CI, 3.46–8.68) and current users (aOR 5.92; 95% CI, 3.73–9.40).

**Conclusions:**

In 2017–2018, HTP use was still limited in Europe among the general population; however, the dual use of these products, their high use among younger generations, and the interest of non-smokers in these products are worrying and indicate the need for close monitoring in terms of prevalence and the characteristics of users.

## INTRODUCTION

Heated tobacco products (HTP) are electronic devices heating a tobacco stick to generate an inhalable aerosol containing nicotine (and other toxicants).^1^ In December 2014, Phillip Morris International (PMI) launched in Italy and Japan its first HTP, named IQOS.^2^^–^^4^ This product is now in commerce in most high-income countries, including the majority of European Union (EU) Member States (MSs). Meanwhile, HTPs from other tobacco companies (eg, Glo by the British American Tobacco and Ploom TECH by Japan Tobacco International) are also available in some EU MSs.

These products raise concerns about their safety.^3^^,^^5^^–^^7^ In fact, although HTPs produce lower levels of some carcinogens and toxic chemicals compared to conventional cigarettes, they are not risk-free.^5^^,^^8^^,^^9^ Moreover, they produce new substances not generated by conventional cigarettes,^7^ having uncertain impact on health.^10^ In addition, long-term effects of HTPs are still unknown.^8^

Besides safety problems, HTPs raise serious public health concerns, similar to those of electronic cigarettes. These include the dual use with other traditional tobacco products and electronic cigarettes of the majority of HTP users, the lack of evidence on the effectiveness of HTPs in assisting smokers to quit, and the possible “gateway effect” due to the use of these products also by non-smokers and young adults who are the target of the tobacco industry and can become addicted to nicotine through HTPs.^11^^–^^17^

Only a few independent studies, mostly from Japan where HTPs are substantially prevalent^4^^,^^8^^,^^18^ and the United States,^19^ have investigated the diffusion and/or the public health consequences of HTPs. To our knowledge, data from Europe are limited to three representative national-based surveys from Italy and United Kingdom showing a limited but increasing prevalence of use,^2^^,^^3^^,^^20^ and the interest in use by non-smokers^3^ and adolescents.^21^ One recent study based on smokers only has identified factors related to product use from six European countries.^22^

The objective of this study was to evaluate HTP spread in Europe, providing the first measure of prevalence and associated factors related to awareness and use of HTPs in 11 European countries.

## METHODS

Within the Horizon2020 - TackSHS project,^23^ we conducted a cross-sectional study, based on a face-to-face survey, in 12 strategically selected European countries: Bulgaria (fieldwork time: October 2017), England (January–February 2017), France (November–December 2017), Germany (June 2018), Greece (June–July 2018), Ireland (November 2017), Italy (November 2016), Latvia (October 2018), Poland (September 2018), Portugal (November–December 2017), Romania (June–July 2017), and Spain (October 2017), representing about 80% of the whole EU population. The detailed methods of the TackSHS survey, including the questionnaire development, have been explained elsewhere.^23^^–^^25^ Briefly, in each country we sampled around 1,000 subjects, representative of the general population aged 15 years or older in terms of age, sex, geographic area, and socioeconomic characteristics. Subjects were selected mainly through a multi-stage or a stratified random sampling. We obtained the approval of the protocol of the TackSHS survey from a local ethics committee in each of the 12 countries, and the protocol was registered inClinicalTrials.gov (ID: NCT02928536).

After providing their consent to participate, all subjects were interviewed by *ad hoc* trained interviewers using computer-assisted personal interviewing (CAPI). The survey used a standardized questionnaire including information on demographic and socioeconomic characteristics, cigarette smoking and electronic cigarette use.^23^^,^^24^ The questionnaire was first developed in English and subsequently translated in country-specific languages by bilingual (ie, local language and English language) tobacco control experts. Thus, questions were asked in participants’ primary languages.

Level of education was categorized as country-specific tertiles of schooling years. Self-assessment of household family economic status relative to the country-specific population was classified into three levels (higher than average, average, and lower than average). All subjects were asked to answer the following question: “Have you ever heard about IQOS (the heat-not-burn tobacco cigarette by Philip Morris International); do you use or did you use it?” (in England, where both IQOS and glo were in the market: “Have you ever heard about heat-not-burn tobacco products; do you use or did you use it?”). Possible answers were: 1) “I have never heard about it”; 2) “I heard about it but I never used it”; 3) “I have just tried it a couple of times”; 4) “I used it in the past (not over the last 30 days)”; 5) “I use it occasionally (5 days or less in the last 30 days)”; 6) “I use it regularly (more than 5 days in the last 30 days)”. Subjects answering options 3–6 were classified as “ever HTP users”, those answering options 5–6 as “current HTP users”.

eTable 1 shows selected country-specific information regarding the commercialization of HTPs (eg, month of debut in the local market). In five countries the survey was conducted less than 12 months after HTP introduction (Bulgaria, England, France, Italy, and Spain) while in six countries it was conducted at least 12 months after HTP introduction in local markets (Germany, Greece, Latvia, Poland, Portugal, and Romania). Moreover, by the time of the survey, no HTP was marketed in Ireland, thus the question on HTPs was not asked in Ireland. Excluding Ireland, the survey included a sample of 10,961 subjects from 11 European countries.

Adjusted odds ratios (aORs) and their corresponding 95% confidence intervals (CIs) for ever HTP use were estimated using unconditional multiple logistic regression models after adjusting for sex, age, level of education, and country. To compute prevalence estimates and aORs we applied a statistical weight to guarantee the representativeness of the general adult population in each country; for pooled estimates we further applied a statistical weight with each country contributing in proportion to its population aged 15 years or over.^26^ Statistical analyses were performed using SAS 9.4 (SAS Institute; Cary, NC, USA).

## RESULTS

Of 10,961 subjects from 11 European countries, 10,839 (98.9%) provided valid responses on HTP awareness and use, and were included in this analysis. Overall, 72.2% had never heard about HTPs (from 53.0% in Greece to 84.6% in Spain), 26.0% had heard about these products but never used them (from 13.4% in Romania to 38.7% in Greece), 1.5% had tried them once or twice, 0.1% were past users and 0.1% were current users (Table 1). Overall, 1.8% of respondents described themselves as ever HTP users (from 0.6% in Spain to 8.3% in Greece). Men were more frequently HTP ever users than women (aOR 1.47; 95% CI, 1.11–1.95). Ever use was inversely related to age (compared with <25 years, aOR 0.84; 95% CI, 0.57–1.22 for 25–44 years; aOR 0.63; 95% CI, 0.43–0.94 for 45–64 years; and aOR 0.14; 95% CI, 0.06–0.32 for ≥65 years of age; *P* for trend <0.001). Ever HTP use was more frequent in ex-smokers (compared with never smokers, aOR 4.32; 95% CI, 2.69–6.95) and current smokers (aOR 8.35; 95% CI, 5.67–12.28), and in electronic cigarette past users (compared with never users, aOR 5.48; 95% CI, 3.46–8.68) and current users (aOR 5.92; 95% CI, 3.73–9.40). No statistically significant relationship was observed between HTP use and socio-economic characteristics, including level of education and household economic status.

**Table 1.** Odds ratios^a^ (OR) for heated tobacco product (HTP) ever vs never use and corresponding 95% confidence intervals (CI) in the European population aged ≥15 years, according to selected individual-level characteristics. TackSHS, 2017–2018

	Total^b^ *N*	Subjects unaware of HTP %	Subjects aware of HTP, %					

			Never tried	HTP ever users				

				Just tried^c^	Past users	Current users	All ever users	

							*N*^b^ (%)	OR (95% CI)
**TOTAL**	10,839	72.2	26.0	1.5	0.1	0.1	268 (1.8)	
**Country (month of fieldwork)**								
Bulgaria (Oct 2017)	1,050	78.7	19.3	1.5	0.2	0.3	21 (2.0)	1.68 (0.58–4.88)
England (Jan–Feb 2017)	1,013	69.1	28.8	1.5	0.4	0.2	21 (2.1)	1.63 (0.92–2.86)
France (Nov–Dec 2017)	1,018	79.1	19.2	1.4	0.2	0.2	18 (1.8)	1.46 (0.83–2.55)
Germany (Jun 2018)	1,012	64.2	34.3	1.3	0.0	0.1	16 (1.4)	1.28 (0.73–2.23)
Greece (Jun–Jul 2018)	1,000	53.0	38.7	6.8	0.5	1.0	83 (8.3)	**6.61 (3.63–12.04)**
Italy (Nov 2016)^d^	1,059	74.7	24.2	1.1	0.0	0.0	10 (1.1)	1
Latvia (Oct 2018)	936	66.4	31.8	1.8	0.1	0.0	17 (1.8)	1.47 (0.19–11.53)
Poland (Sep 2018)	724	63.7	34.7	1.6	0.1	0.0	11 (1.6)	1.36 (0.71–2.58)
Portugal (Nov–Dec 2017)	1,000	64.5	32.5	2.5	0.0	0.5	30 (3.0)	**2.57 (1.16–5.70)**
Romania (Jun–Jul 2017)	1,001	83.6	13.4	2.6	0.4	0.0	30 (3.0)	**2.61 (1.35–5.02)**
Spain (Oct 2017)	1,026	84.6	14.7	0.6	0.0	0.1	11 (0.6)	0.53 (0.24–1.18)
**Sex**								
Women	5,727	76.5	22.1	1.2	0.1	0.2	109 (1.4)	1
Men	5,112	67.5	30.4	1.9	0.2	0.1	159 (2.1)	**1.47 (1.11–1.95)**
**Age**, years^e^								
15–25	1,350	69.0	28.3	2.3	0.4	0.0	42 (2.7)	1
25–44	3,699	65.9	31.8	1.9	0.1	0.2	154 (2.3)	0.84 (0.57–1.22)
45–64	3,922	71.7	26.7	1.5	0.1	0.1	66 (1.7)	**0.63 (0.43–0.94)**
≥65	1,838	86.8	12.9	0.2	0.0	0.1	6 (0.3)	**0.14 (0.06–0.32)**
*P* for trend								**<0.001**
**Education** ^f^								
Low	4,136	76.8	21.7	1.3	0.1	0.1	68 (1.5)	1
Medium	3,799	70.4	27.8	1.6	0.1	0.2	107 (1.8)	0.99 (0.70–1.40)
High	2,901	67.8	30.0	1.7	0.3	0.2	93 (2.2)	1.30 (0.91–1.86)
*P* for trend								0.166
**Household economic status** ^f^								
Lower than average	2,711	78.1	20.4	1.1	0.3	0.1	49 (1.5)	1
Average	5,624	74.4	23.9	1.5	0.1	0.1	150 (1.7)	0.99 (0.66–1.48)
Higher than average	1,394	68.3	29.0	2.3	0.2	0.3	51 (2.8)	1.61 (0.99–2.63)
*P* for trend								0.060
**Smoking status**								
Never smokers	5,828	76.7	22.8	0.4	0.0	0.1	26 (0.5)	1
Ex-smokers	1,886	70.8	27.2	1.8	0.1	0.1	41 (2.0)	**4.32 (2.69–6.95)**
Current smokers	3,125	63.2	32.5	3.7	0.5	0.3	201 (4.4)	**8.35 (5.67–12.28)**
**Electronic cigarette status** ^f^								
Never users	10,295	73.1	25.5	1.2	0.1	0.1	194 (1.4)	1
Past users	291	54.9	36.2	7.1	0.8	1.0	42 (8.9)	**5.48 (3.46–8.68)**
Current users	247	56.1	34.9	6.5	0.9	1.6	32 (8.9)	**5.92 (3.73–9.40)**

CI, confidence interval; HTP, heated tobacco products; OR, odds ratio.

^a^ORs and their corresponding 95% CIs were estimated using unconditional multiple logistic regression models adjusting for sex, age, level of education, and country. Estimates in bold are statistically significant at 0.05 level.

^b^Unweighted number of subjects with available information on HTP use (*N* = 10,839) or unweighted number of ever users of HTPs (*N* = 268).

^c^Subjects reporting to have tried HTP a couple of times.

^d^Italy was used as the pilot country, thus, its fieldwork was anticipated to November 2016. Italy has been chosen as the reference category because it is the first European country where IQOS was launched.

^e^Participants’ age was ≥15 years in all countries, except England (≥16), Ireland (≥18), Greece (15–64), and Latvia (15–74).

^f^The sum does not add up to the total because of few missing values. In particular, 3 subjects had missing information on level of education and 6 subjects had missing information on electronic cigarette use. Moreover, the variable on self-assessment of the household (family) economic status relative to the country-specific population was missing in all German participants (*n* = 1,012) plus further 98 subjects.

In countries where HTPs were introduced less than 12 months before the survey conduction, the prevalence of HTP awareness was 23.3% and that of HTP ever users was 1.4%. Corresponding estimates in countries where HTPs were introduced more than 12 months before the survey conduction were 34.3% and 2.2% (*P* < 0.001 and *P* = 0.001 for awareness and use, respectively).

Among 41 HTP ever users and ex-cigarette smokers at the time of the survey, 20 (49%) quit smoking before HTPs were introduced in the local market of each country.

## DISCUSSION

This study provides the prevalence and factors related to the HTP use among the general population in multiple European countries. We found that the prevalence of ever HTP use (and the frequency of use) was still low. Overall, almost 2% of European adults already tried (or used) HTPs, with a wide variation between countries. Young people, men, ever smokers, and electronic cigarette users were more likely to be attracted to these new products, in line with previous researches.^21^^,^^27^ The large variation in HTP awareness (ranging between 15% and 47%) and use (ranging between 1% and 8%) also reflects differences in the commercialization of these products. In fact, countries with a shorter experience of HTPs (ie, with time since HTPs introduction in the local market lower than 12 months at the time of the survey) were those with a lower awareness and use of these tobacco products.

Subjects aged 15–24 years have more frequently tried HTPs, in agreement with previous findings.^15^^,^^20^ This suggests that the youngest generations could be the key target subpopulation of HTP marketing promotions. However, the number of current HTP users among the young is null, likely due to the relatively expensive device—as compared to electronic cigarette—that discourages initiation for a regular use.^17^

Although no statistically significant relationship has been observed between the use of HTP and socioeconomic characteristics, wealthier subjects systematically reported the highest levels of HTP awareness and use, in line with previous studies.^3^^,^^20^

The majority of HTP ever users were dual users (ie also smokers of conventional cigarettes). This raises concerns that, similar to what has been observed for electronic cigarettes,^28^^–^^30^ HTPs could also be used in places where combustible cigarettes are not permitted while HTPs are not covered by the national legislation. These findings are in line with a recent research conducted among 6,027 smokers from 6 EU MSs according to which ever use of HTPs was significantly higher among those who had tried to quit smoking in the last 12 months, had tried electronic cigarettes during their lifetime and among those that perceived HTPs as less dangerous than combustible cigarettes.^22^ A qualitative study conducted in the United Kingdom in 2018 found that IQOS users mostly experimented with IQOS to reduce or to stop smoking combustible cigarettes due to health risks, perceiving IQOS as an (healthier) alternative way to continue smoking.^17^ Moreover, only a few had completely stopped smoking both IQOS and combustible cigarettes, as people mainly used IQOS in place of (or alongside) traditional cigarettes.^17^

In line with the current evidence,^3^ our study found that a limited but not negligible proportion of HTP users (ie, 10% of all HTP ever users) were never smokers, suggesting that these products are attracting towards nicotine dependence new segments of population, with potentially different characteristics from those known to be associated with exclusive tobacco consumption. Moreover, almost half of ex-smokers who used HTPs quit smoking before HTPs were launched in the local markets. This supports the fact that a large proportion of ex-smokers using HTPs are not people switching to HTPs to reduce their harm, but people who relapse nicotine addiction, being attracted by this new alleged safe product. How HTPs are attracting non-smokers to nicotine addiction is a matter of concern that requires further research.

This study has some weaknesses, mainly related to its cross-sectional design. Although the overall sample size is large, our study is based on a small sample of European HTP users. In many countries the survey was conducted only a few months after HTP introduction into the market, and the fieldwork was conducted in different time periods (ranging between November 2016 and October 2018), making the comparisons of HTP use among countries difficult. Furthermore, some differences in terms of sampling methodology used^24^ add complexity to the interpretation of the results. Moreover, no pictures of heated tobacco products were shown to participants during the interviews. This, together with some possible differences in the wording of questions asked in various countries, may have resulted in a response bias. The strengths of our survey include the representativeness of the adult population of the included European countries in terms of age, sex and habitat, and the use of face-to-face interview. More importantly, this is the first European study evaluating the spread of HTP among the general population, using the same standardized questionnaire in different countries. Therefore, our estimates may serve as key baseline measures for future studies.

Our study shows that only 0.1% of Europeans currently use HTPs. Once generalized to the 28 EU MSs, our estimate is compatible with more than half million adults currently using this product. Our data show that HTPs are: i) mainly used in combination with other products, ii) mainly used by the youngest generations, and iii) also used by (and likely promoted to) never smokers. These findings are worrying and indicate the need for close monitoring of prevalence, trends and determinants of HTP use. Our study could be useful to support the planning, implementation and monitoring of targeted prevention intervention, as well as inform decision making at the normative and public health level in Europe.

## APPENDIX A. SUPPLEMENTARY DATA

eTable 1. Information on heated tobacco products (HTP) commercialization in 12 European countries
