# Supplementary material for: Use and Awareness of Heated Tobacco Products in Europe
Source: J Epidemiol. 2022 Mar 5;32(3):139–44. doi: 10.2188/jea.JE20200248 (PMC8824661; doi:10.2188/jea.JE20200248)
Supplement: Supplementary file 1 [file je-32-139-s001.pdf]

eTable 1. Information on heated tobacco products (HTP) commercialization in 12 European countries

Country	Fieldwork date	Date of launch of HTPs	Time since HTPs introduction in the local market[a]	HTPs available at the time of the survey
Bulgaria	Oct 2017	Nov 2017[b]	1 month[b]	IQOS
England	Jan–Feb 2017	Dec 2016	2 months	IQOS
France	Nov–Dec 2017	Apr 2017	7 months	IQOS, glo[c]
Germany	Jun 2018	Jun 2016	24 months	IQOS
Greece	Jun–Jul 2018	March 2017[d]	15 months	IQOS
Italy	Nov 2016	Dec 2015[e]	11 months	IQOS
Ireland	Nov 2017	Not in commerce	-	-
Latvia	Oct 2018	Jun 2016	28 months	IQOS
Poland	Sep 2018	May 2017	16 months	IQOS
Portugal	Nov–Dec 2017	Nov 2015	24 months	IQOS
Romania	Jun–Jul 2017	Nov 2015	19 months	IQOS
Spain	Oct 2017	Dec 2016	10 months	IQOS

[a] Difference between the date of launch oh HTPs and the date of the conduction of the fieldwork, in each country.

[b] IQOS was introduced in the Bulgarian market in November 2017, but the introduction of IQOS was discussed in the media at the beginning of October 2017.

[c] glo products have been on the French market between end-2017 and end-2019, and were withdrawn later on.

[d] In October 2016, IQOS was introduced in Athens. Only in March 2017 the market was extended to the whole Greece.

[e] In December 2014, IQOS was introduced in Milan. Only in December 2015 the market was extended to the whole Italy.
